# Hostels for Unmarried Mothers

**Published:** 1920-06-12

**Authors:** 


### Hostels for Unmarried Mothers.

THE Marchioness of Titchfield has issued an appeal for help to start a hostel for unmarried mothers, as advocated by the National Birthrate Commission. The Margaret Club and Day Nursery Committee (Ampthill Square), propose to organise one in connection with their crèche for fatherless children, and the scheme is to start a residential club at which each mother will have her own bed-sitting room where she can live with her child, either providing her own meals, or taking them in a club room. It is also proposed to have a sick room for babies where minor ailments can be treated, so that mothers will not be prevented from carrying on their work. The Marchioness of Titchfield calculates that £1,000 will be required to set it going, and hopes that once it is established it may be almost self-supporting.

